# Modified halloysite nanotube filled polyimide composites for film capacitors: high dielectric constant, low dielectric loss and excellent heat resistance†

**DOI:** 10.1039/c8ra01373j

**Published:** 2018-03-15

**Authors:** Tianwen Zhu, Chao Qian, Weiwen Zheng, Runxin Bei, Siwei Liu, Zhenguo Chi, Xudong Chen, Yi Zhang, Jiarui Xu

**Affiliations:** PCFM Lab, GD HPPC Lab, Guangdong Engineering Technology Research Centre for High-performance Organic, Polymer Photoelectric Functional Films, State Key Laboratory of Optoelectronic Materials and Technologies, School of Chemistry, Sun Yat-sen University Guangzhou 510275 China ceszy@mail.sysu.edu.cn +86 20 84112222 +86 20 84112222

## Abstract

In this work, halloysite nanotubes (HNTs) were chosen as the fillers and high performance polyimide (PI) as the matrix to form a series of dielectric composite materials with high dielectric constant, low dielectric loss and excellent heat resistance. Firstly, KH550 was used to modify the surface of HNTs to make sure of a good dispersion of HNTs into the polymer. The results showed that the addition of KH550 modified HNTs (K-HNTs) can improve the dielectric constant of the composite films while maintaining their excellent dielectric loss properties. To further increase the dielectric constant of the HNTs/PI composites, conductive polyaniline (PANI) was used to coat the surface of HNTs to obtain PANI modified HNTs (PANI-HNTs). Compared with the K-HNTs filled systems, the dielectric constant of the PANI-HNTs/PI nanocomposite films is greatly enhanced. The highest dielectric constant of the PANI-HNTs/PI films can achieve 17.3 (100 Hz) with a low dielectric loss of 0.2 (100 Hz). More importantly, the as-prepared composite films have high breakdown strengths (>110.4 kV mm^−1^) and low coefficients of thermal expansion, as low as 7 ppm per °C, and a maximum discharge energy density of 0.93 J cm^−3^. Also, such properties are maintained stably up to 300 °C, which is critical for manufacturing heat-resisting film capacitors.

## Abstract 2

Halloysite nanotubes (HNTs) were chosen as the fillers and polyimide (PI) as the matrix to form a series of composites with excellent dielectric properties and thermostabilities.

## Graphical abstract

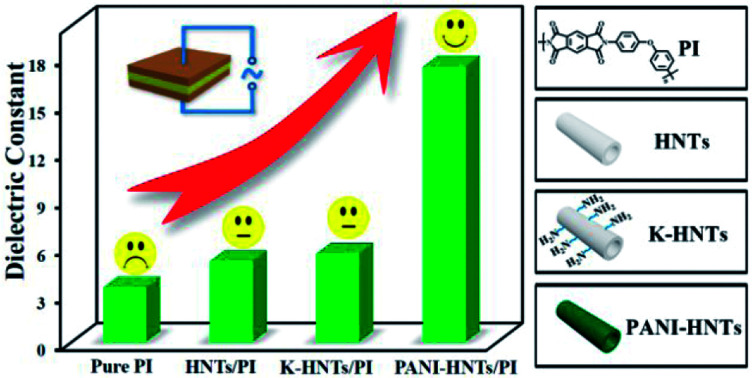

## Introduction

Capacitors are a kind of passive component which is widely used in modern electronic and electrical industries owing to their fast charge and discharge capabilities.^1–3^ There are mainly five types of capacitors, which are film capacitors, ceramic capacitors, mica capacitors, electrolytic capacitors and electrochemical capacitors.^4^ Among them, film capacitors based on polymers show a great promise for developing flexible and wearable electronic devices in the future^5,6^ due to their flexibility, easy processing, low cost and excellent dielectric properties. However, the dielectric constant of most of the polymers (usually *k* < 4) is too low to obtain capacitors with high capacitance and discharge energy density.^7–9^ On the other hand, the commercialized high dielectric polymer materials, for example polyvinylidene fluoride (PVDF) and its copolymer P(VDF-CTFE) (*k* > 8), are still limited by their low operating temperature (<175 °C).^10–13^ Therefore, for the manufacture of heat-resisting film capacitors used for extreme conditions, like aerospace power system, automobile and underground oil exploration, excellent thermostable polymeric materials with high dielectric constant and low dielectric loss are desirable.

Among numerous polymer materials, polyimide (PI) is considered as the best candidate for these film capacitors on account of its high glass transition temperature, brilliant flexibility and outstanding resistance to solvents.^14^ Though PI films possess low dielectric losses (0.001–0.03), their low dielectric constants are frustrated. In general, there are two approaches for achieving higher dielectric constant for polyimide materials. One is to fill high-*k* ceramic fillers, such as TiO_2_,^15^ Al_2_O_3_,^16^ BaTiO_3_ (ref. 17–20) and CaCu_3_Ti_4_O_12_,^21–23^ into the polyimide matrix. For example, Dang *et al.*^24^ added calcium copper titanate (CCTO) into the polyimide for composite films. Thanks to the giant dielectric constant (∼10^4^) of CCTO, the composite film exhibited high dielectric constant (∼49) when the concentration of fillers reached 40 vol% at 10^2^ Hz, while low dielectric loss (<0.2, 100 Hz) and other admirable properties were still maintained. Unfortunately, the dielectric loss would dramatically increase to 1.2 at 10^2^ Hz when the test temperature reached 150 °C, which was too high for practical applications. In addition, Wang *et al.*^25^ used *in situ* dispersive polymerization for preparing BaTiO_3_ nanowires/polyimide (BT-NWs/PI) and BaTiO_3_ nanoparticles/polyimide (BT-NPs/PI) composites with low volume fraction (≤10 vol%) of ceramic fillers. Compared with the BT-NPs/PI composites, they unexpectedly found that the dielectric constant and energy storage density of the composites was enhanced by using BT-NWs at the same concentration. Nevertheless, getting a high dielectric constant demanded a large amount of ceramic fillers (>67 wt%), which might cause serious agglomeration problem and reduce flexibility of the composite films. Besides, a high temperature sintering (>1000 °C) was required for preparing ceramic materials. The other approach for raising the *k* value of polyimide materials is to introduce electrically conductive fillers, such as graphene oxide (GO),^26^ or carbon nanotubes (CNTs),^27–29^ into the polymer matrix based on the percolation theory.^30,31^ The key issue of this strategy is to produce microcapacitor networks at the percolation threshold to acquire a distinct enhancement of dielectric constants. Dang *et al.*^32^ prepared a series of silver/polyimide composite films through a simple *in situ* polymerization with an ultra-high dielectric constant (400, 10^3^ Hz) when the volume fraction of silver particles reached 12.5%. However, high level of dielectric loss (more than 1 at 10^3^ Hz) hindered its application. To solve the above problems sufficiently, modified conductive fillers/polymer composites were emerged.^33^ For instance, Liu *et al.*^34^ reported their sandwich-like SiO_2_ coated graphene oxide hybrids/polyimide composites. As the weight fraction of the fillers reached 10%, a dielectric constant of 20 (40 Hz) was obtained while the dielectric loss was controlled below 0.1 (40 Hz). Obviously, this pathway for high-*k* composites avoids large contents of fillers, but still leads to a corresponding decrease of breakdown strength and is too costly for industrial production.

Halloysite nanotubes (HNTs) are naturally occurring clay minerals which have shown great potential usages in biotechnology,^35^ substances adsorption/separation,^36,37^ energy storage/conversion,^38^ catalysis^39^ and sustained release of chemical agents^40–42^ in recent years due to their unique tubular structures. Halloysite is consist of chemical formula of Al_2_Si_2_O_5_(OH)_4_·2H_2_O, which is resemble to kaolinite except for the presence of an additional water monolayer between the adjacent clay layers.^43–46^ Compared with carbon nanotubes, halloysite nanotubes are economically available materials and possess many unique characteristics, including different outside/inside chemical properties and moderate hydroxyl groups on the surface for chemical modification.^47^ Furthermore, some monomers can be easily induced to form polymer on the external surface because of their high specific area and distinctive charge distribution.^48–54^ Thus, halloysite nanotubes are described as reinforcing materials for polymers in mechanical properties^55^ but less concerned on dielectric properties. The dielectric constant of halloysite nanotubes is as high as 6–8 with an extremely low dielectric loss of 10^−3^. Therefore, through proper surface modification, HNTs might be an ideal reinforcement for preparing dielectric polymer composite materials with high dielectric constant and low dielectric loss properties.

In this work, halloysite nanotubes (HNTs) were chosen as the fillers and high performance polyimide (PI) as the matrix to prepare a series of dielectric composite materials with high dielectric constant, low dielectric loss and excellent heat resistance. Firstly, KH550 were used to modify the surface of HNTs to make sure the well dispersion of HNTs into the polymer. The addition of KH550 modified by HNTs (K-HNTs) does improve the dielectric constant of the composite films, while maintain their excellent dielectric loss properties. To further increase the dielectric constant of the HNTs/PI composite, conductive polyaniline (PANI) was used to coat onto the surface of HNTs to get PANI modified HNTs (PANI-HNTs). The dielectric constant of the PANI-HNTs/PI nanocomposite films is greatly enhanced. The highest dielectric constant of the PANI-HNTs/PI films was 17.3 (100 Hz) with a low dielectric loss of 0.2 (100 Hz). More importantly, the as-prepared composite films own high breakdown strengths (>110.4 kV mm^−1^) and low coefficients of thermal expansion (as low as 7 ppm per °C), and the maximum discharge energy density of 0.93 J cm^−3^. It has been found that such properties are maintained stable up to 300 °C, exceeding all the reported polymeric dielectrics in heat resistance, and are absolutely critical for the manufacturing of heat-resisting film capacitors.

## Experimental

### Materials

Halloysite nanotubes (HNTs) were purchased from Imerys Tableware Asia Limited. Concentrated sulfuric acid (H_2_SO_4_), ethanol and acetic anhydride were analytical grade reagents received from Guangzhou Chemical Reagent Co., Ltd. Aniline, 3-aminopropyl triethoxysilane (KH550), ammonium persulfate (APS), pyromellitic anhydride (PMDA) and 4,4′-oxydianiline (ODA) were all obtained from Aladdin. *N*,*N*′-Dimethylacetamide (DMAc, water ≤ 50 ppm) was purchased from Energy Chemical Co., Ltd. All the reagents were used without further purification.

### Surface modification of HNTs using KH550

KH550 modified HNTs were prepared using a method reported previously.^56^ Typically, 5.0 g HNTs were dispersed in 100 ml of a mixture of 95 : 5 (v/v) ethanol/water using an ultrasonic processor and then stirring for 2 h. Meanwhile, 4 ml KH550 was hydrolyzed for 1 h in the mixture of 0.8 ml water and 10.2 ml ethanol, the pH of which was adjusted to 4–5 by acetic anhydride. Afterwards, the hydrolyzed KH550 was added to the slurry and stirred for another 20 h at 120 °C. Later the slurry were vacuum filtered, washed with deionized water for several times and dried at 80 °C for 12 h. The products were named K-HNTs.

### Surface modification of HNTs using polyaniline

Polyaniline modified HNTs were synthesized by one-step *in situ* polymerization of aniline on the surface of HNTs. Considering the later thermal imidization process, the high boiling sulphuric acid was chosen as the doped acid, while ammonium persulfate (APS) was used as an oxidant. In a typical reaction, 1.5 g HNTs, 1 ml aniline and 9.0 ml H_2_SO_4_ were mixed into 250 ml water. The mixtures were ultrasonic irradiated and then stirred for 1 h at room temperature. At the same time, 4.0 g APS was dissolved in 100 ml water (containing 0.7 ml H_2_SO_4_) with magnetic stirrer for 30 min to obtain a homogeneous solution.

Next, the solution was added dropwise into the colloidal mixtures within 30 min with magnetic stirring at 2.5 °C and then kept stirring for 18 h. After the polymerization, the dark green products were centrifugated and washed by water for several times until neutral. Finally, the products were dried under vacuum at 80 °C overnight and named PANI-HNTs.

### Preparation of HNTs/PI and modified HNTs/PI composite films

Firstly, a series of HNT/PAA, K-HNTs/PAA and PANI-HNTs/PAA solutions were prepared by sonication and magnetic stirring, the concentrations of HNTs, K-HNTs and PANI-HNTs ranged from 0 to 40 wt%, 0 to 40 wt% and 0 to 50 wt%, respectively. The specific steps were as follows: HNTs or modified HNTs (including K-HNTs and PANI-HNTs) were dispersed in DMAc under ultra-sonication and magnetic stirring for 24 h. After that, the diamine monomer ODA was completely dissolved to get a homogeneous solution. PMDA was then added into the solution and stirred for 20 min to gain a sticky polyamic acid (PAA) solution. The fillers (HNTs or modified HNTs) were introduced into the PAA solution with a solid content of 10–12 wt%. All these mixtures were stirred at room temperature for 8 h.

Secondly, the viscous HNT/PAA, K-HNTs/PAA and PANI-HNTs/PAA gluesolution were cast onto clean glass plates and then thermal imidized in a vacuum oven by a three-step imidization protocol: 100 °C/1 h, 200 °C/1 h and 350 °C/1 h.

**Fig. 1:**
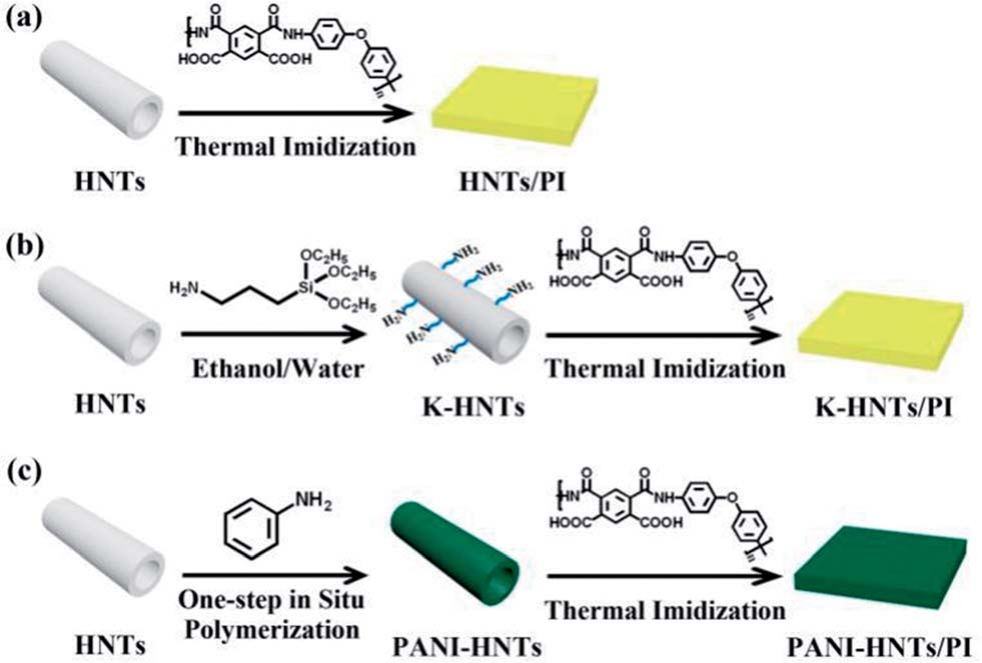
Synthetic routs of the HNTs/PI composites. (a) The HNTs/PI composite film; (b) K-HNTs/PI composite film; (c) PANI-HNTs/PI composite film.

Finally, the composite films were obtained and the thickness of which was 30–40 μm. These complete synthetic routes are shown in Fig. 1.

### Characterization

Elemental analysis (EA) of C, H and N was performed on Elementar vario EL instrument. Fourier-transform infrared spectra (FT-IR) for fillers and attenuated total reflection infrared (ATR-IR) spectra for composite films were both recorded by a Bruker Tensor 27 spectrometer. The crystal structure of fillers was characterized by X-ray diffraction (XRD, Rigaku D-Max) with a Cu-Kα radiation (*λ* = 1.5418 Å). Transmission electron microscopy (TEM) images were obtained from a Tecnai G2 Spirit instrument at an accelerating voltage of 120 kV. The cross-sectional morphologies of the composites films were investigated by scanning electron microscopy (SEM, Hitachi S-4800) at an accelerating voltage of 10 kV. Thermo gravimetric analyses (TGA) were carried out using a PE Pyris 1 thermo gravimetric analyzer at a heating rate of 10 °C min^−1^ with a nitrogen flow of 20 ml min^−1^. To get the coefficients of thermal expansion, TA TMA Q400 machine was employed for thermal mechanical analysis. In addition, frequency dependent dielectric properties were conducted by Solartron SI 1260 Impedance/Gain Phase Analyzer together with two copper electrodes (10.2 × 10.2 mm^2^). Meanwhile, silver paste was coated onto both side surfaces of the samples to ensure excellent contact between the electrodes and samples (Fig. S1 in the ESI†). The frequencies of the tests were ranged from 100 Hz to 1 MHz at room temperature or others adjusted by a hot plate. The breakdown strengths were conducted by a programmable withstanding voltage tester CS9915AX (Nanjing Chansheng Instrument Co., Ltd.) 5 times on each specimen for accuracy.

## Results and discussion

### Characterization of HNTs and modified HNTs

**Fig. 2:**
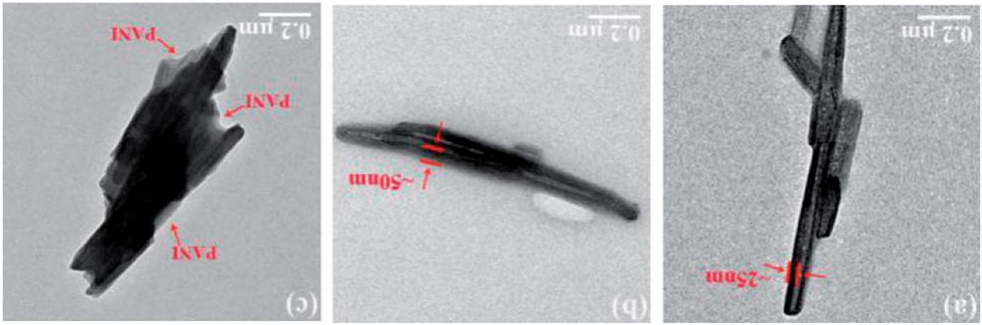
TEM images of (a) HNTs, (b) K-HNTs and (c) PANI-HNTs.

The naturally occurring halloysite nanotubes are chosen as the fillers for fabricating inorganic/polymer composites owning to their unique chemical properties. Although HNTs are difficult to reunite spontaneously comparing with other nanoparticles, their low surface hydroxyl density may still be harmful to the uniform distribution in the polymer matrix. As for polyimide composites, amino-modification exhibits great significance for fillers on account of their chemical coupling with the polymer matrix. Consequently, silane coupling agent and polyaniline were used for surface modification, which might be beneficial for improving the dispersibility of fillers and the dielectric properties of the resulted composites. The detailed morphological features of HNTs, K-HNTs and PANI-HNTs were investigated by TEM. As shown in Fig. 2a, the original HNTs possess lumen and external diameters about 20–30 nm and 70–80 nm, respectively, while their lengths can reach 0.5–1.5 μm. The TEM images (Fig. 2b and c) provide direct evidence for the successful surface modification. Comparing with the original HNTs, K-HNTs own thicker tube walls and PANI-HNTs are covered with obvious polyaniline layers which result in a rougher surface. Besides, we accidentally find that the interaction between polyaniline layer and HNTs are quite strong so that even ultrasonic washing cannot remove the coated layers from the surface.

In the previous studies, Li *et al.*^45,57,58^ had demonstrated that the negative charge would appear on the outer surface of HNTs when the pH was below 2, which could induce polyaniline to *in situ* grow at low temperature owning to the electrostatic interaction between anilinium cation (=NH_2_^+^) and negatively charged surface. The specific procedure can be explained by the *in situ* polymerization mechanism of aniline on solid substrates: at the initial polymerization stage, oligomers are formed and trend to be adsorbed onto the surface of HNTs; these oligomers are considered to act as nucleation sites for growing polyaniline chains. Thus, once a polyaniline chain grows on the surface, it will promote more new polyaniline chains to surround the HNTs.

The XRD pattern of original HNTs is shown in Fig. S2 in the ESI,† where strong diffraction peaks at 2*θ* of 12.0°, 19.9°, 24.5°, 34.9°, 39.5°, 54.5°, 62.3°, 73.6° and 77.0° can be seen, which are attributed to the crystal plane of (001), (100), (002), (110), (003), (210), (300), (220) and (310), respectively. Compared with original HNTs, K-HNTs display almost the same XRD pattern, whereas things become quite different for PANI-HNTs. As a result of doping H_2_SO_4_, the crystal structures of HNTs are partial destroyed and have a little bit tendency to transfer towards amorphous structures. Furthermore, a broad peak observed from 22.0° to 30.0°, which belongs to amorphous polyaniline according to the literatures.^59,60^

**Fig. 3:**
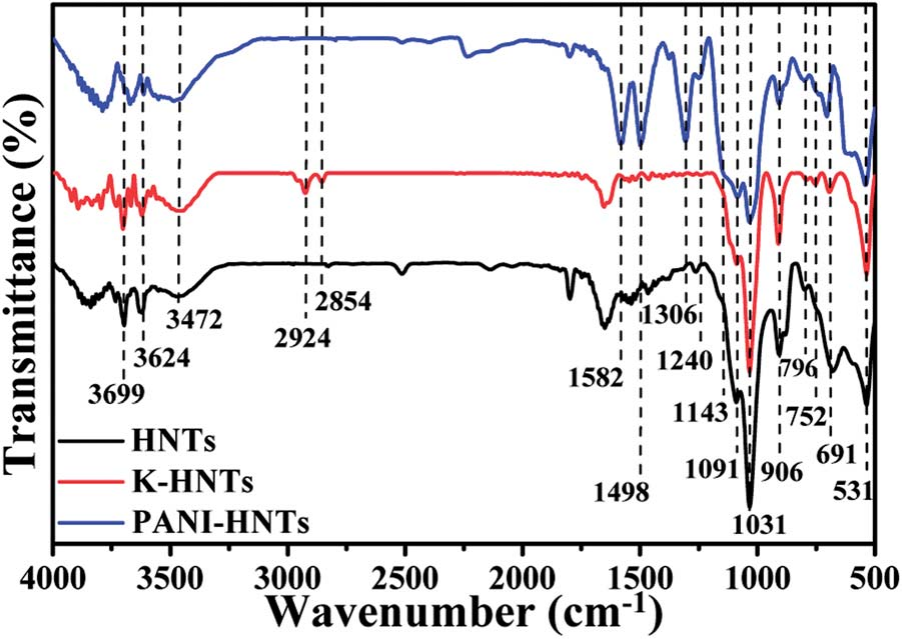
FT-IR spectra of HNTs, K-HNTs and PANI-HNTs.

Fig. 3 shows the FT-IR spectra of HNTs, K-HNTs and PANI-HNTs. As for the original HNTs, the characteristic peaks at 3699 cm^−1^, 3624 cm^−1^ are corresponded to Al_2_OH stretching and the peak at 906 cm^−1^ is assigned to the single Al_2_OH bending, which proves the existence of alumina layers. A board peak appears at 3500–3200 cm^−1^ range with a maximum at 3472 cm^−1^ is ascribed to SiO–H vibration, which is from the silica layers. In addition, the peaks at 1091 cm^−1^, 1031 cm^−1^ and 796 cm^−1^ are attributed to the Si–O–Si plane bending vibration, Si–O stretching and Si–O–Si stretching, respectively. The signals at 752 cm^−1^, 691 cm^−1^ and 531 cm^−1^ are associated with the Si–O–Al in plane bending, which reflects the combination of two layers. In contrast to the original HNTs, K-HNTs exhibit two new peaks at 2924 cm^−1^ and 2854 cm^−1^ corresponding to the CH stretching vibration and the CH_2_ stretching vibration. Besides, several distinct peaks can be discovered for the existence of polyaniline: the peaks of 1582 cm^−1^ and 1498 cm^−1^ are assigned to C=C stretching vibration in quinonoid unit and C=C stretching vibration in benzenoid unit, respectively; the peaks of 1306 cm^−1^ and 1240 cm^−1^ are, individually, ascribed to C–N and C–N^+^ stretching vibration in benzenoid units; the peak may be ignored at 1143 cm^−1^ is regard as =N^+^– stretching vibration in the doped quinonoid unit.^61,62^ Additionally, the exact contents of modifiers (KH550, polyaniline) can be affirmed by the C, H, N elemental analysis shown in Table 1.

**Table 1:** The C, H, N elemental analysis of HNTs, K-HNTs and PANI-HNTs

Sample	C (%)	H (%)	N (%)	SUM (%)
HNTs	—	1.333	—	1.333
K-HNTs	2.38	2.161	0.65	5.191
PANI-HNTs	16.21	3.353	2.99	22.553

### Characterization of HNTs/PI and modified HNTs/PI composites

**Fig. 4:**
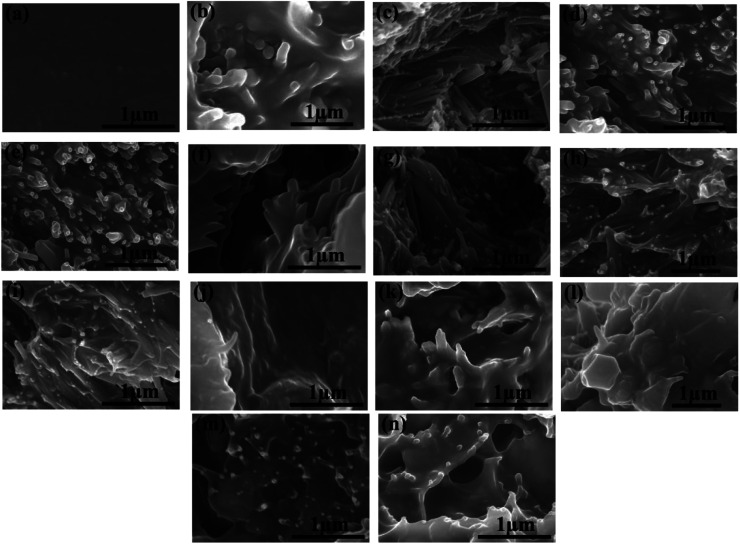
Cross-sectional SEM images of (a) pure PI; HNTs/PI composite films: (b) 10 wt%, (c) 20 wt%, (d) 30 wt%, (e) 40 wt%; K-HNTs/PI composite films: (f) 10 wt%, (g) 20 wt%, (h) 30 wt%, (i) 40 wt% and PANI-HNTs/PI composite films: (j) 10 wt%, (k) 20 wt%, (l) 30 wt%, (m) 40 wt%, (n) 50 wt%.

To explore the dispersion of HNTs and modified HNTs in the PI matrix, the composite films were dipped into liquid nitrogen and then broken. The cross-sectional SEM images of the pure PI and the PI composites with filler contents various from 0 to 40 wt% or 0 to 50 wt% are shown in Fig. 4.

It can be observed from Fig. 4a that the pure PI film is dense without obvious pores. Considering the composite films, superb compatibilities between fillers and polymer matrix are achieved when the weight fractions of fillers are below 20%. For HNTs/PI films with higher contents of fillers, the polyimide matrix fails to wrap fillers up uniformly (Fig. 4d and e). However, after facile surface modification, K-HNTs and PANI-HNTs show super-duper adhesion to polyimide matrix (Fig. 4f–n). Especially for the PANI-HNTs/PI system, even at a high content, the PANI-HNTs are still surrounded by matrix equably because of the potential entanglement between polyimide and polyaniline chains. As a result, PANI-HNTs can be loaded at a higher weight fraction (50%) comparing with the other two systems (40%).

The photographs of composite films are presented in Fig. S3 (see ESI†) exhibiting excellent flexibility which is beneficial for devices manufacture.

The chemical compositions of the as-prepared composite films were determined by FT-IR spectra (Fig. S4 in the ESI†). Evidently, there is no obvious absorption at 3500–3300 cm^−1^ (–NH_2_ stretching), meanwhile the characteristic peaks of the imide groups at 1778 cm^−1^, 1716 cm^−1^ (symmetrical stretching of carbonyl), 1369 cm^−1^ (stretching of C–N) and 720 cm^−1^ (bending of carbonyl) appeared, indicating complete imidization is achieved. In addition, the characteristic peaks of HNTs at 1027 cm^−1^ (plane bending of Si–O–Si), 913 cm^−1^ (single bending of Al_2_OH) and 681 cm^−1^ (in plane bending of Si–O–Al) strengthen when the weight fraction of fillers increase, whereas the peaks of polyaniline at 1592 cm^−1^ (stretching of C=C in quinonoid unit) and 1498 cm^−1^ (stretching of C=C in benzenoid unit) change little.

### Thermal properties

The thermal properties of the fillers and the composite films were examined by TGA and TMA. As shown in Fig. S5a,† there are mainly two weight losses stages in the degradation of HNTs. The first stage is below 100 °C, which corresponds to the loss of absorb water in the internal and external surface of HNTs. The second stage between 500 °C and 600 °C is assigned to the dehydroxylation process to remove all the interlaminar crystal water according to the previous report.^38^ Besides, the primary weight loss difference of K-HNTs and HNTs is caused by the degradation of the silane coupling agent starting from 400 °C to 800 °C. In the case of PANI-HNTs, compared with HNTs, the TGA curve shows two more weight-loss stages, 200–400 °C and over 600 °C, which are considered as the thermal decomposition of oligomers and polymer backbone, respectively.^63^

The TGA and DTG results of composite films are listed in Table S1 (ESI) and Fig. S5 (ESI†). Distinctly, all these films exhibit great thermal stability with insignificant weight loss up to 500 °C in nitrogen and degradation of polyimide backbone happen around 700 °C. The 5% and 10% weight-loss temperature of pure polyimide are 665 °C and 683 °C, respectively. When the weight fraction of fillers is controlled within 10%, the heat resistance of composites will improve unexpectedly, 10 wt% PANI-HNTs/PI in particular, exhibiting the highest 5 wt% and 10 wt% weight-loss temperature at 668 °C and 703 °C, respectively. Nevertheless, with the increase of filler contents, composites will emerge the characteristic weight-loss stages of fillers, which lower the 5 wt% and 10 wt% weight-loss temperature of composites comparing with pure PI.

**Fig. 5:**
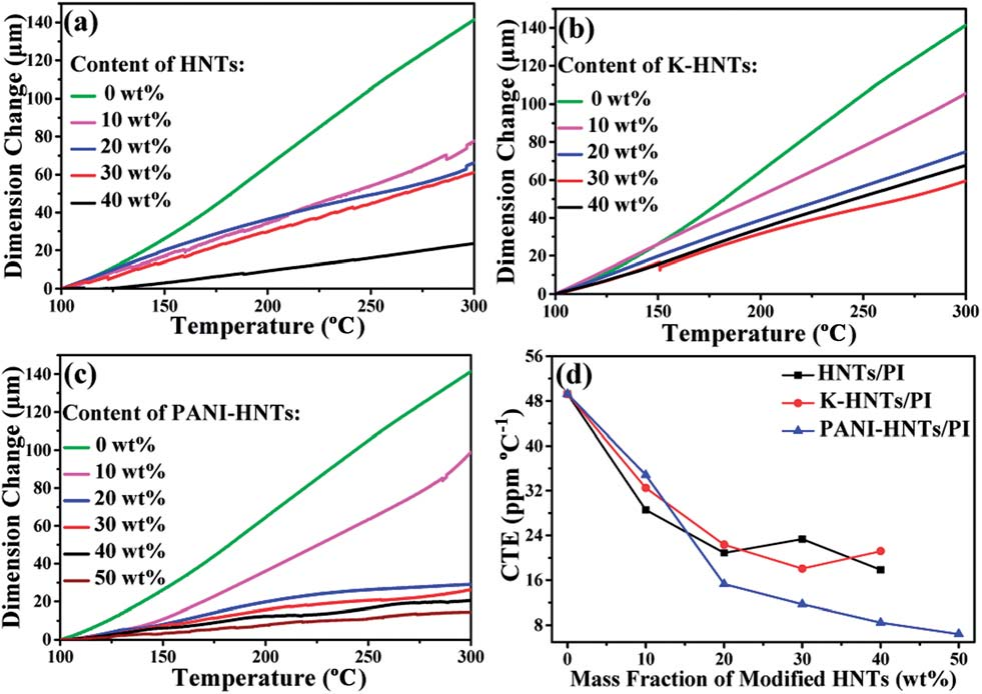
Linear thermal expansion (a–c) and coefficients of thermal expansion (d) of HNTs/PI, K-HNTs/PI and PANI-HNTs/PI composite films measured from 100–300 °C.

To fabricate film capacitors using in high temperature conditions, the coefficients of thermal expansion (CTE) for the dielectric films and metal electrodes need to be matched well, otherwise the capacitors will disintegrate. Commonly, the CTE values of polymer films are too high to match with metal, such as copper, aluminum and others. Accordingly, it is crucial to lower the CTE values of polyimide films by incorporating inorganics with superior dimensional stabilities, HNTs and modified HNTs in particular, are believed to be the most promising candidates for preparing composites with low CTE values. As shown in Fig. 5, the CTE values of the composites decrease with the fillers contents. When the weight fraction of HNTs or modified HNTs reaches 40 wt%, the CTE values of composite films drop to around 20 ppm per °C, which are able to match with copper (17.5 ppm per °C), silver (19.5 ppm per °C) and aluminum (23 ppm per °C) perfectly, comparing with the original PI films (49 ppm per °C). Accidently, we find that the 50 wt% PANI-HNTs/PI shows the lowest CTE value at 7 ppm per °C. This result may be also explained by the possible entanglement between polyimide and polyaniline chains, which strengthens the molecular interaction and contributes to enhance the dimensional stability of the composites.

### Dielectric properties

The capacitance of a plate capacitor is defined as the equation:

1 *C* = *ε*_0_*εS*/*d*

where *ε*_0_ is the vacuum permittivity (*ε*_0_ = 8.85 × 10^−12^ F m^−1^), *ε* is the permittivity of dielectric material; *S* and *d* represent the areas and distances of polar plates, respectively. Moreover, for the linear dielectrics, the maximum discharge energy density can be calculated as the equation:

2 *U*_e_ = 1/2*ε*_0_*εE*_B_^2^

where the breakdown strength (*E*_B_) is investigated.^7–9^ These two equations prove that dielectric constant plays a crucial role in achieving capacitors with high capacitance and discharge energy density.

**Fig. 6:**
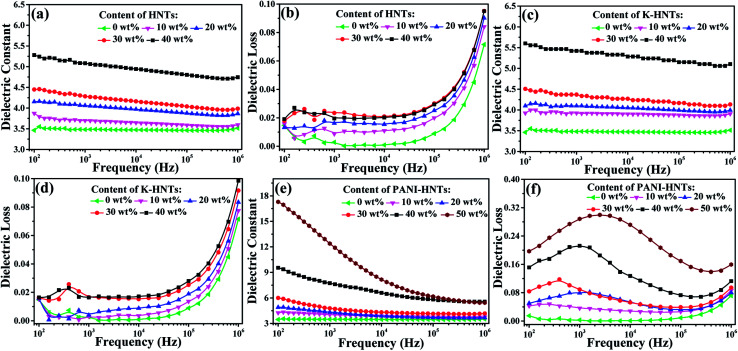
Frequency dependence of the (a) dielectric constant, (b) dielectric loss (tan *δ*) of HNTs/PI composite films; (c) dielectric constant, (d) dielectric loss (tan *δ*) of K-HNTs/PI composite films; (e) dielectric constant, (f) dielectric loss (tan *δ*) of PANI-HNTs/PI composite films measured at room temperature from 100 Hz to 1 MHz.

The frequency dependent dielectric constant and dielectric loss of the composite films with various loading levels of fillers are studied over a frequency range of 100 Hz to 1 MHz at room temperature, and the results are shown in Fig. 6. Apparently, with the addition of HNTs and K-HNTs, the dielectric constant of the composites increases as expected while the dielectric loss remains quite low. It is particularly noteworthy that after mixing 40 wt% fillers into the polyimide matrix, the dielectric constant of HNTs/PI and K-HNTs/PI can reach 5.28 and 5.60 at 100 Hz, respectively. More surprisingly, the dielectric loss of these two composites is able to be controlled below 0.02 and closed to pure PI, which is never reported before. Furthermore, the dielectric loss increases abruptly from 10^5^ Hz to 10^6^ Hz can be attributed to the dielectric relaxation behaviors of polyimide. For the PANI-HNTs/PI films, the dielectric constant and dielectric loss were enhanced with the amount of fillers increase. However, unlike HNTs/PI and K-HNTs PI, they exhibit strong frequency dependence when the weight fraction of fillers exceeds 20% due to interfacial polarization under external electric field. The maximal dielectric constant found for the PANI-HNTs/PI composites is 17.3, with the filler content of 50 wt%, as compared with 3.5 for that of pure PI at 100 Hz.

According to the previous theories, the local displacement of charge stored in dielectrics would occur under external electric field, which was considered as polarization phenomenon. Ordinarily, there are four types of polarization in dielectrics: electronic, atomic, orientational (or dipolar) and space charge (electrons/holes and ions) polarizations.^64^ Electronic and atomic polarizations belong to the resonance regime, and they happen in the optical (10^14^ to 10^16^ Hz) and infrared frequencies (10^11^ to 10^14^ Hz), respectively. Besides, orientational polarization, taking place in 10^4^ to 10^11^ Hz, is able to induce the dielectric dipole turning to the direction of external electric field. Unlike the others, space charge polarization occurs below 10^4^ Hz owning to nonuniform distribution of space charge rather than dielectric itself. Space charge polarization can be further divided into hopping polarization (in the bulk) and interfacial polarization (at interfaces).^65^

In our work, the test frequencies of dielectric properties are ranged from 10^2^ Hz to 10^6^ Hz, so that the mainly polarization behaviors occurred for polyimide composites are orientational and interfacial polarization. With the incorporation of HNTs or modified HNTs, strong chemical bonding is constructed between the matrix and fillers to impede free movement of polyimide chains and lead them to grow radially. Therefore, as comparison to pure PI, dipolar redistribution happens on HNTs/PI or modified HNTs/PI composites, which brings about considerable enhancement of dielectric constants above 10^4^ Hz (Fig. 6a, c and e).

The Maxwell–Wagner Effect pointed out that the mechanism of interfacial polarization was accounted for charge accumulation at the two-material interface on the basis of the difference of charge carrier relaxation times in these two materials.^65^

**Fig. 7:**
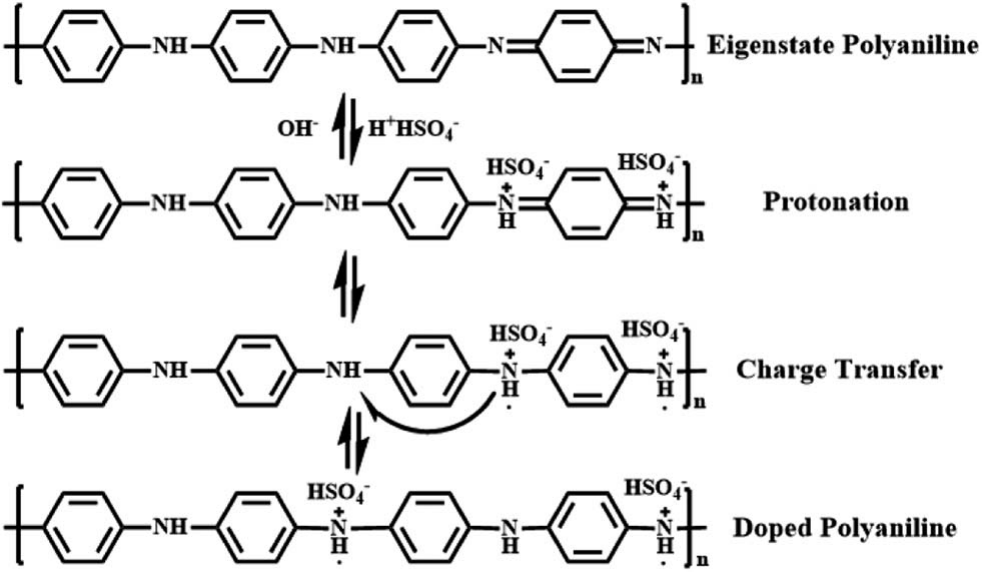
Detailed formation procedures for doped polyaniline.

Polyaniline (PANI) is found to be the most promising intrinsically conducting polymers (ICPs) because of its ease of synthesis, low cost monomer, tunable properties and better stability compared to other ICPs. When the eigenstate polyaniline is doped with H_2_SO_4_, hydrogen ions will firstly protonate the nitrogen atoms to form bipositive species (quinonoid unit). But these species are highly precarious and perform strong tendency on transferring to more stable polaron structure (benzenoid unit) with extra electrons, which is responsible for charge storage and electrical conduction through hopping mechanism.^62^ All these procedures are shown in Fig. 7 in detail.

Except for orientational polarization happening in high frequencies (10^4^ to 10^6^ Hz), PANI-HNTs/PI composites also exhibit strong interfacial polarization. Once the doped PANI-HNTs are exposed to external electric field, a large amount of charge will be accumulated at the interface between fillers and polyimide matrix, which leads to variation of macroscopic dipole moment and giant improvement of dielectric properties at a certain loading level (>20 wt%) in low frequencies (<10^4^ Hz). Both of these two polarized phenomena contribute to enhance dielectric constant while unavoidable increase of dielectric loss occurs.

Fig. S6 (ESI†) shows that the dielectric constants of all the composites were enhanced when increasing the contents of fillers, and an obvious percolation phenomenon displays on PANI-HNTs/PI composites while the percolation threshold is over 50 wt%. Such a percolation phenomenon can be ascribed to the semiconductor property of the PANI-HNTs from AC conductivity shown in Fig. S7.† Remarkably, enhancements of AC conductivity occur in the PANI-HNTs/PI composites while the HNTs/PI and K-HNTs/PI composites are roughly consistent with pure PI. It is noteworthy that all the modified HNTs/PI composites keep prominent insulation as dielectrics. Besides, after facile surface modification by KH550, K-HNTs show strong interaction with the polyimide matrix which results in improvement of dielectric constants and slight decrease of dielectric losses as compared with those of HNTs/PI. Although the dielectric loss of 50 wt% PANI-HNTs/PI composites reaches 0.2, they are still available for film capacitors.

**Fig. 8:**
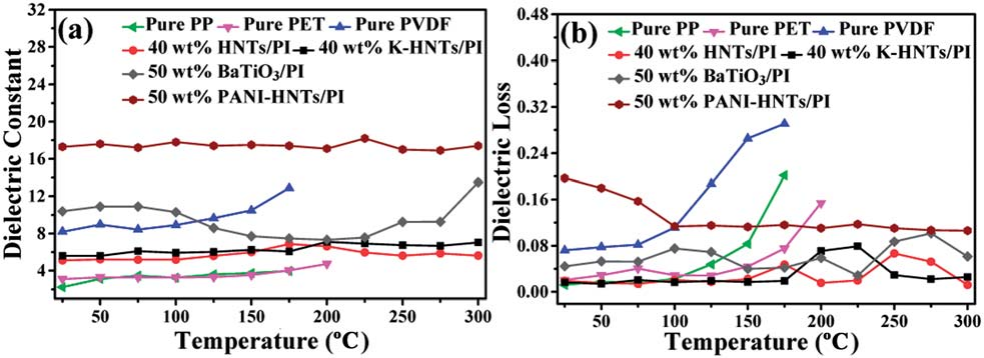
Temperature dependence of the (a) dielectric constant, (b) dielectric loss (tan *δ*) of pure PP, pure PET, pure PVDF, 40 wt% HNTs/PI, 40 wt% K-HNTs, 50 wt% BaTiO_3_/PI and 50 wt% PANI-HNTs/PI composite films measured in 100 Hz.

Based on the foregoing statement, polyimide was chosen as the matrix because of its inherent stability facing some extreme conditions, such as elevated temperature, intense radiation and high humidity. Hence, temperature dependent dielectric properties of composite films are urgently needed and the results are plotted in Fig. 8.

As comparison, the temperature dependent dielectric properties of commercial dielectrics (polypropylene (PP), polyethylene glycol terephthalate (PET) and polyvinylidene fluoride (PVDF)) were also examined. Apparently, these commercial dielectrics exhibit poor thermal stability owning to their low melting points or softening temperature. When the ambient temperature reached close to their melting or softening points, the destruction of films started with exaggerated rises in dielectric losses (Fig. 8b). However, polyimide composites in this work (40 wt% HNTs/PI, 40 wt% K-HNTs/PI and 50 wt% PANI-HNTs/PI) show superb heat resistance to retain steady dielectric constants even though the environmental temperature is up to 300 °C. Besides, for the purpose of comparison we also synthesized 50 wt% BaTiO_3_/PI composites samples using the same methods in our work. It is observed that two clear undulations of dielectric constant happen when test temperature reach 100 °C and 300 °C, which are corresponding to the two crystalline transformations of BaTiO_3_, respectively. Therefore, HNTs/PI or modified HNTs/PI composites are more competitive in comparison with commercial or frequently-used dielectrics.

### Breakdown strengths and energy storage properties

It is clear that the maximum discharge energy density is decided by the *ε* and *E*_B_ values of dielectrics, *E*_B_, in particular, is a pivotal factor since *U*_e_ has a quadratic dependence on the *E*_B_. Consequently, the way to enhance the dielectric constant while maintaining high breakdown strength of the polymer films is considered as the most effective strategy to develop materials with high discharge energy density. Commonly, the breakdown strengths of composites obey the two-parameter Weibull distribution function:^9^

3 *P* = 1 − exp[−(*E*/*E*_B_)^*β*^]

where *P* is the cumulative probability of electrical failure, *E* is experimental breakdown strength, the characteristic breakdown strength (*E*_B_) is obtained when the cumulative probability of electrical failure (*P*) equaled 63.2% and *β* is the shape parameter calculated through linear fitting. This distribution function can convert into its logarithmic form:

4 log[−ln(1 − *P*)] = *β*(log *E* − log *E*_B_)

when the value of log[−ln(1 − *P*)] reaches zero, the *E* is equal to *E*_B_. Besides, for every specific value of *E*, *P* is calculated as follows:

5 *P* = (*i* − 0.44)/(*n* + 0.25)

where *i* indicates that this *E* value ranks the ith in the ascending order of breakdown strength data and *n* is the number of total data points.^66^

**Fig. 9:**
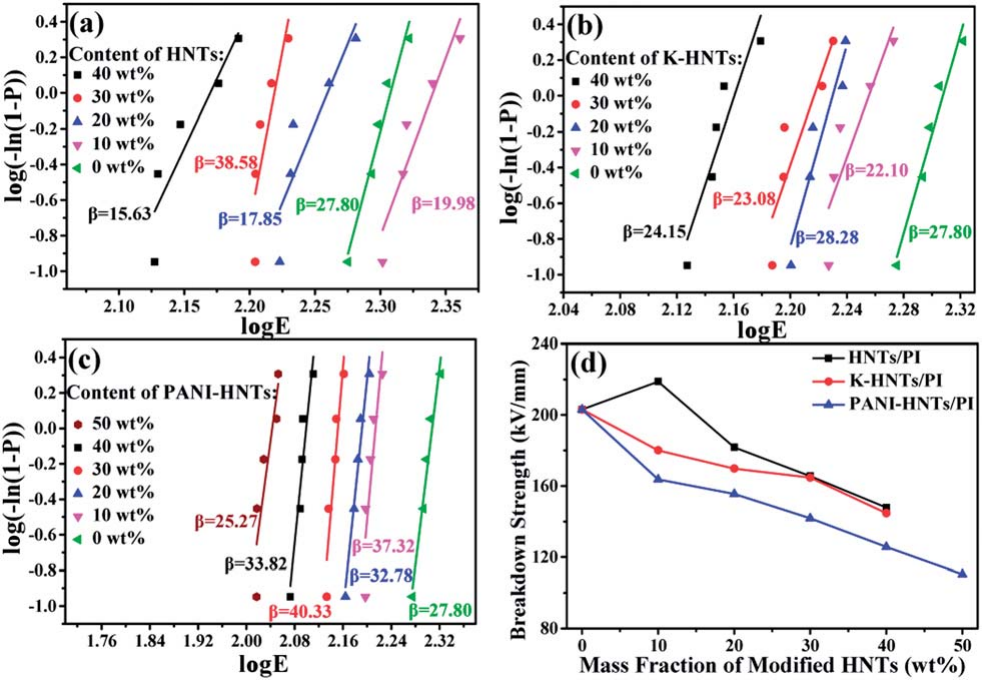
Weibull-distribution plots of breakdown strength for (a) HNTs/PI; (b) K-HNTs/PI and (c) PANI-HNTs/PI composite films. (d) The breakdown strengths of composites.

In Fig. 9, all the HNTs/PI or modified HNTs/PI composite films show high *β* values, indicating high-quality and stability of the composites. The breakdown strengths (*E*_B_) of these composite films with various fillers loading are shown in Fig. 9d. Generally, the breakdown strengths decrease distinctly with increasing the contents of fillers owning to the destruction of insulation gradually taken place. However, the abnormal phenomenon happens on 10 wt% HNTs/PI composite film, which can be explained as follows: the polyimide matrix fails to wrap HNTs up uniformly and heterogeneous distribution of fillers occurs. As a result, the 10 wt% HNTs/PI composite film will be divided into multilayers, which are conducive to develop breakdown strengths under electric field. Furthermore, this phenomenon disappears when the contents of fillers exceed above 20 wt% due to the increase of dielectric constants. The breakdown strength of pure PI film is quite high (203.1 kV mm^−1^). Although large amounts (≥40 wt%) of HNTs, K-HNTs and PANI-HNTs in the polyimide matrix may partially damage the insulation of composites, the breakdown strengths can still be kept at 147.9 kV mm^−1^, 144.7 kV mm^−1^ and 110.4 kV mm^−1^, respectively. To our best of knowledge, for the reported high-*k* polyimide composites, like CNTs/PI, the breakdown strengths would sharply fall below 100 kV mm^−1^ with a low content of fillers (10 wt%).^33^ Thus, by keeping the insulation of composites, our strategy obtains a series of dielectrics with high breakdown strengths, which are advanced in manufacturing devices suffering from high voltage.

**Fig. 10:**
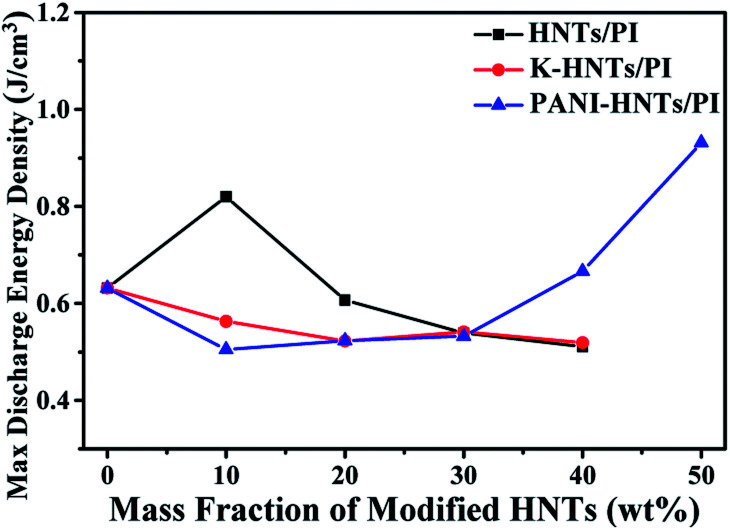
Max discharge energy density of composites at room temperature (100 Hz).

As discussed above, the maximum discharge energy density of linear dielectrics is dependent on the dielectric constants and breakdown strengths. In Fig. 10, thanks to the high breakdown strength, 10 wt% HNTs/PI composite film exhibits a maximum discharge energy density up to 0.82 J cm^−3^. The highest maximum discharge energy density is obtained for the 50 wt% PANI-HNTs/PI composite film at 0.93 J cm^−3^, which promotes over 50% than that of pure PI (0.63 J cm^−3^).

## Conclusions

In summary, halloysite nanotubes (HNTs) were chosen as the fillers and high performance polyimide (PI) as the matrix to form a series of new dielectric composite materials. The dispersibility of KH550 modified HNTs in the polyimide matrix was improved significantly as compared with original HNTs. Both the HNTs/PI and modified HNTs/PI composites show not only enhanced dielectric constants but also decreased CTE values with increasing loading of fillers. When HNTs were coated with conductive polyaniline (PANI-HNTs), the dielectric constant of PANI-HNTs/PI composite films can be further enhanced. The 50 wt% PANI-HNTs/PI composite film possesses the highest dielectric constant of 17.3 at 100 Hz with a low dielectric loss of 0.2 (100 Hz), better than other reported HNTs based nanocomposites,^67^ and the maximum discharge energy density could of 0.93 J cm^−3^. More importantly, these composite films show prominent breakdown resistance and are able to work at 300 °C steadily, which may be explained in terms of the orientational and interfacial polarizations. This study paves a facile way for expanding applicability of polyimide composites as high-performance dielectric materials for high-temperature electronic and energy devices, particularly for the manufacture of heat-resisting film capacitors.

## Conflicts of interest

There are no conflicts to declare.

## Supplementary Material

RA-008-C8RA01373J-s001
