# Supplementary material for: Modified halloysite nanotube filled polyimide composites for film capacitors: high dielectric constant, low dielectric loss and excellent heat resistance
Source: RSC Adv. 2018 Mar 15;8(19):10522–31. doi: 10.1039/c8ra01373j (PMC9078885; doi:10.1039/c8ra01373j)
Supplement: RA-008-C8RA01373J-s001 [file RA-008-C8RA01373J-s001.pdf]

Electronic Supplementary Material (ESI) for RSC Advances.
This journal is © The Royal Society of Chemistry 2018

Supporting Information

Modified Halloysite Nanotubes filled Polyimide Composites for Film Capacitors: High Dielectric Constant, Low Dielectric Loss and Excellent Heat Resistance

Tianwen Zhu, Chao Qian, Weiwen Zheng, Runxin Bei, Siwei Liu, Zhenguo Chi, Xudong Chen, Yi Zhang* and Jiarui Xu

PCFM Lab, GD HPPC Lab, Guangdong Engineering Technology Research Centre for High-performance Organic and Polymer Photoelectric Functional Films, State Key Laboratory of Optoelectronic Materials and Technologies, School of Chemistry, Sun Yat-sen University, Guangzhou 510275, China.

List of Contents for Supplementary Materials:

Fig. S1 Detailed procedure to fabricate film capacitors for testing dielectric properties. 2

Fig. S2 XRD patterns of HNTs, K-HNTs and PANI-HNTs 2

Fig. S3 Photographs of (a) pure PI; HNTs/PI composite films: (b) 10 mt%, (c) 20 mt%, (d) 30 mt%, (e) 40 mt%; K-HNTs/PI composite films: (f) 10 mt%, (g) 20 mt%, (h) 30 mt%, (i) 40 mt% and PANI-HNTs/PI composite films: (j) 10 mt%, (k) 20 mt%, (l) 30 mt%, (m) 40 mt%, (n) 50 mt% 3

Fig. S4 FT-IR spectra of (a) HNTs/PI, (b) K-HNTs/PI and (c) PANI-HNTs/PI composite films... 3

Table S1 The thermal properties of composite films with varied mass fractions. 4

Fig. S5 TGA and DTG curves (inset) of (a) fillers; (b) HNTs/PI, (c) K-HNTs/PI and (d) PANI-HNTs/PI composite films.. 4

Fig. S6 (a) The dielectric constant and (b) dielectric loss (tan δ) of HNTs/PI, K-HNTs/PI and PANI-HNTs/PI composite films measured at room temperature (100 Hz)............................. 5

Fig. S7 The AC conductivity of (a) HNTs/PI, (b) K-HNTs/PI and (c) PANI-HNTs/PI composite films.. 5

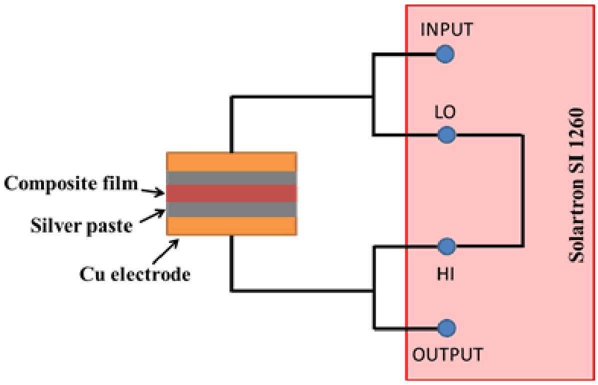

Fig. S1 Detailed procedure to fabricate film capacitors for testing dielectric properties.

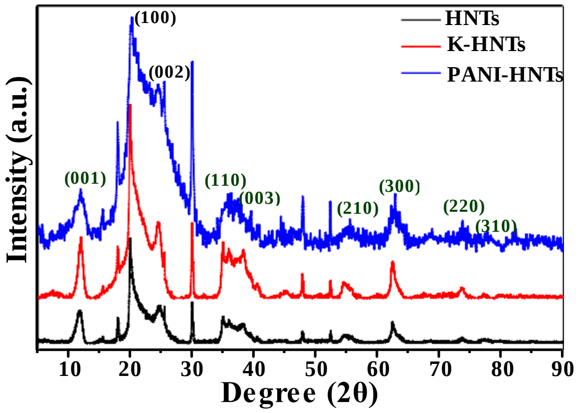

Fig. S2 XRD patterns of HNTs, K-HNTs and PANI-HNTs.

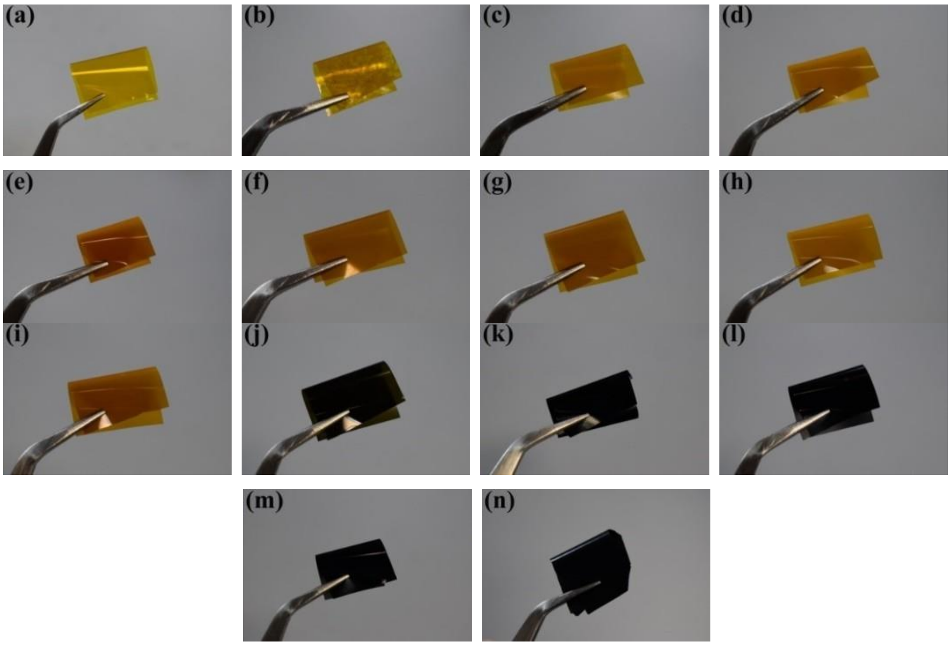

Fig. S3 Photographs of (a) pure PI; HNTs/PI composite films: (b) 10mt%, (c) 20mt%, (d) 30mt%, (e) 40mt%; K-HNTs/PI composite films: (f) 10mt%, (g) 20mt%, (h) 30mt%, (i) 40mt% and PANI-HNTs/PI composite films: (j) 10mt%, (k) 20mt%, (l) 30mt%, (m) 40mt%, (n) 50mt%.

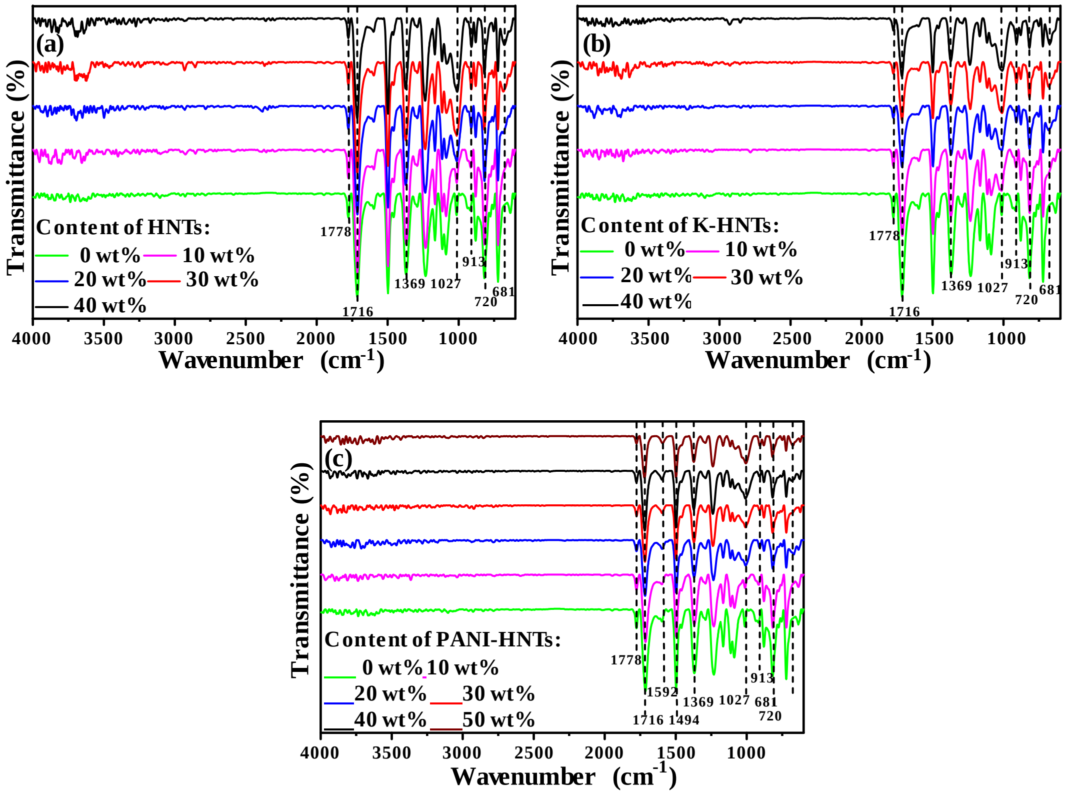

Fig. S4 FT-IR spectra of (a) HNTs/PI, (b) K-HNTs/PI and (c) PANI-HNTs/PI composite films.

Table S1 The thermal properties of composite films with varied mass fractions.

Sample	$T_{d5\%}$ (°C)	$T_{d10\%}$ (°C)	Sample	$T_{d5\%}$ (°C)	$T_{d10\%}$ (°C)
Pure PI	665	683	30 wt% K-HNTs/PI	592	681
10 wt% HNTs/PI	659	697	40 wt% K-HNTs/PI	587	684
20 wt% HNTs/PI	612	686	10 wt% PANI-HNTs/PI	668	703
30 wt% HNTs/PI	609	685	20 wt% PANI-HNTs/PI	570	687
40 wt% HNTs/PI	612	684	30 wt% PANI-HNTs/PI	595	686
10 wt% K-HNTs/PI	659	698	40 wt% PANI-HNTs/PI	538	674
20 wt% K-HNTs/PI	596	687	50 wt% PANI-HNTs/PI	535	653

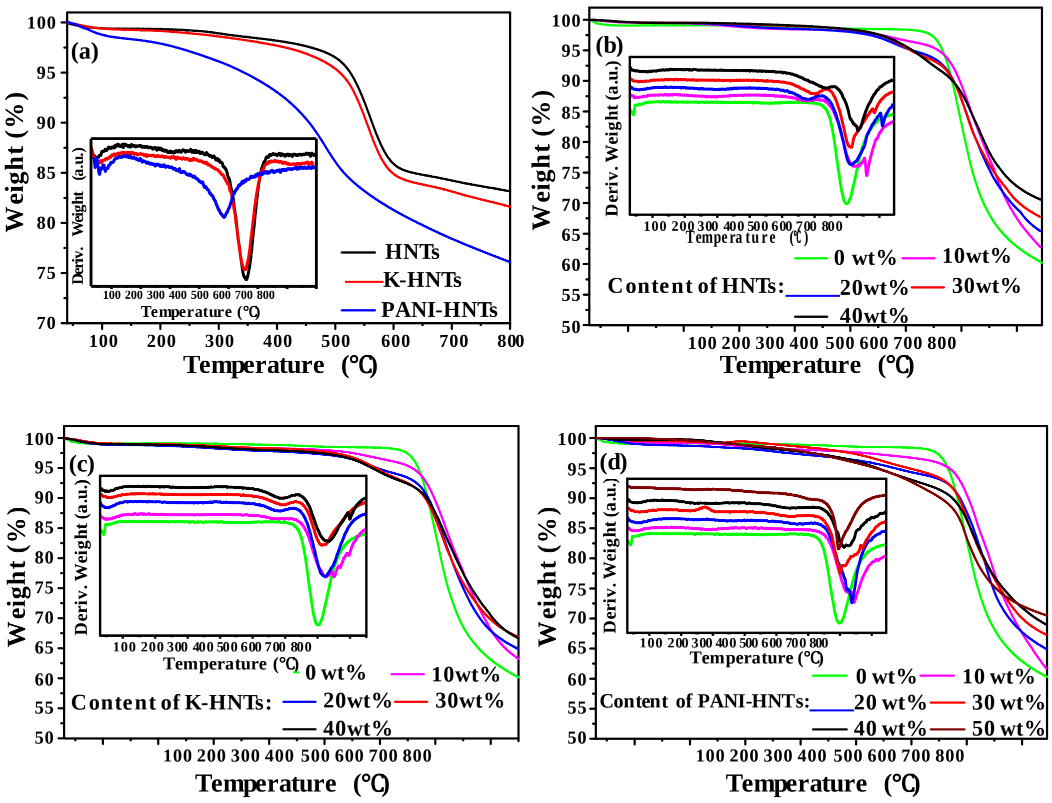

Fig. S5 TGA and DTG curves (inset) of (a) fillers; (b) HNTs/PI, (c) K-HNTs/PI and (d) PANI-HNTs/PI composite films.

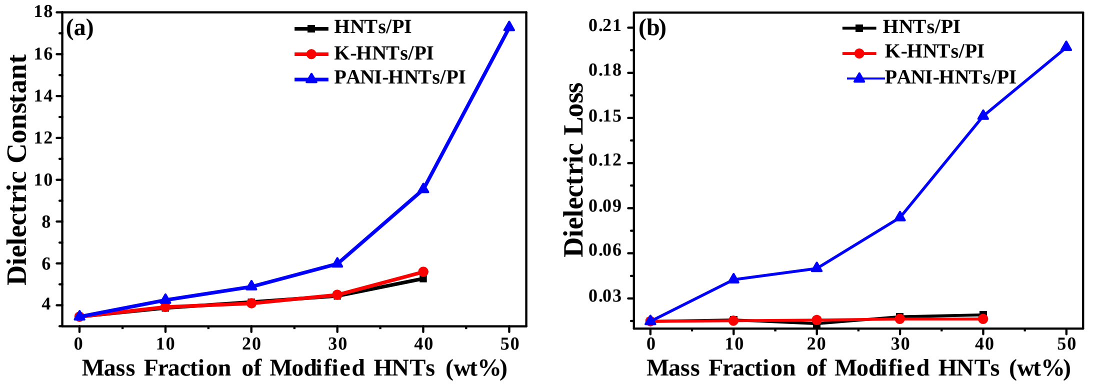

Fig. S6 (a) The dielectric constant and (b) dielectric loss (tan δ) of HNTs/PI, K-HNTs/PI and PANI-HNTs/PI composite films measured at room temperature (100 Hz).

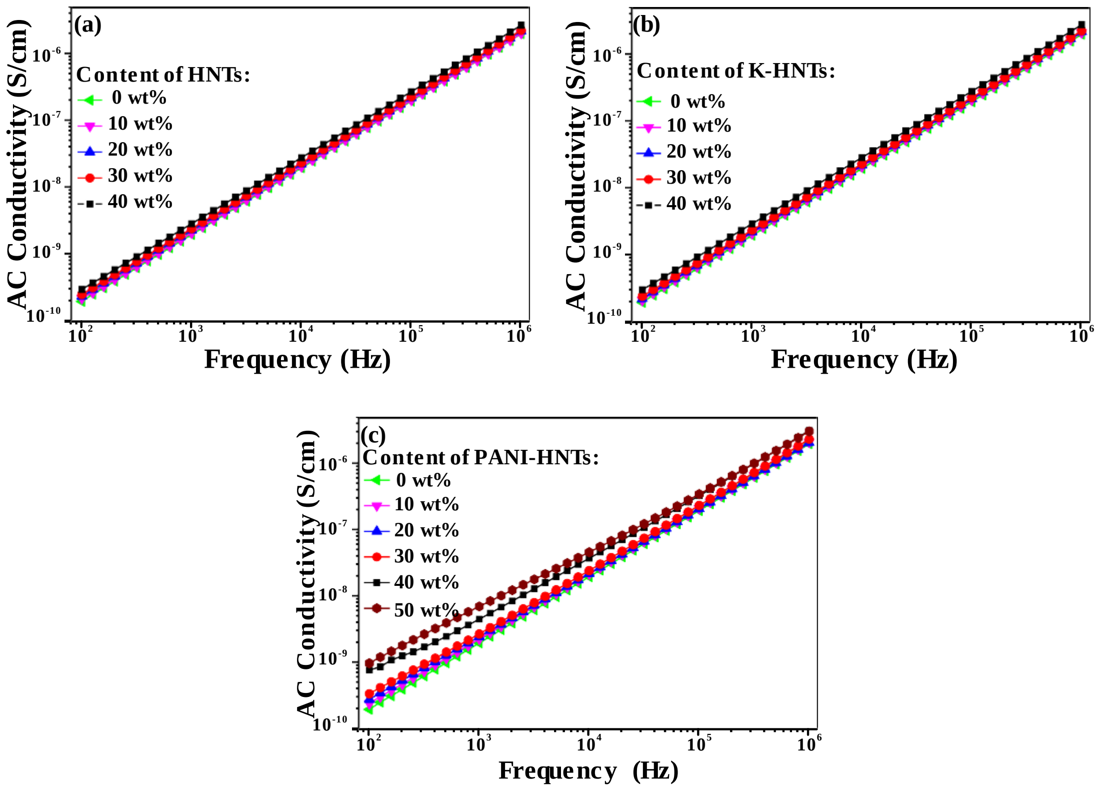

Fig. S7 The AC conductivity of (a) HNTs/PI, (b) K-HNTs/PI and (c) PANI-HNTs/PI composite films.
